# A global dataset to parametrize critical nitrogen dilution curves for major crop species

**DOI:** 10.1038/s41597-022-01395-2

**Published:** 2022-06-07

**Authors:** Ignacio Ciampitti, Emmanuela van Versendaal, Juan Francisco Rybecky, Josefina Lacasa, Javier Fernandez, David Makowski, Gilles Lemaire

**Affiliations:** 1 Department of Agronomy, Kansas State University, Manhattan, Kansas US; 2 University Paris-Saclay, INRAE, AgroParisTech, UMR MIA 518, 75231 Paris, France; 3 Honorary Director of Research, INRAE, 86600 Lusignan, France

**Keywords:** Plant physiology, Natural variation in plants

## Abstract

Precise management of crop nitrogen nutrition is essential to maximize yields while limiting pollution risks. For several decades, the critical nitrogen (N) dilution curve - relating plant biomass (W) to N concentration (%N) - has become a key tool for diagnosing plant nutritional status. Increasing number of studies are being conducted to parameterize critical N dilution curves of a wide range of crop species in different environments and N-fertilized conditions. A global synthesis of the resulting data is lacking on this topic. Here, we conduct a systematic review of the experimental data collected worldwide to parametrize critical N dilution curves. The dataset consists of 36 papers containing a total of 4454 observations for 19 major crop species distributed in 16 countries. The key variables of this dataset are the W and %N collected at three or more sampling times, containing three or more fertilizer N rate levels. This dataset can guide the development of generic critical N dilution curves, helps scientists to identify factors influencing plant N status, and leads to the formulation of more robust N recommendations for a broad range of environmental conditions.

## Abstract (summary)

**Table:** 

Measurement(s)	biomass • N concentration
Technology Type(s)	field data collection • field data collection and lab analysis
Factor Type(s)	nitrogen nutrition index
Sample Characteristic - Organism	field crops
Sample Characteristic - Environment	agricultural systems
Sample Characteristic - Location	global dataset

## Background & Summary

Nitrogen (N) is one of the most limiting factors for agricultural productivity, and mineral N fertilizers represent a key input for many cropping systems worldwide^1^. However, the over-application of N fertilizers to field crops has a significant impact on the environment through release of greenhouse-gas emissions and air pollutants^2,3^, groundwater pollution, and eutrophication of freshwater^4^ and marine ecosystems^5^. Increasing N use efficiency could reduce the footprint of N fertilization in agricultural systems while ensuring a high level of production. This goal could be achieved through more precise N rate recommendations and a better application schedule adapted to the crop N requirements.

Defining the optimal N fertilization is a daunting task, due to the large uncertainties in predicting soil N supply^6^, plant growth and N demand^7^. Nitrogen fertilization is often either insufficient (under high fertilizer prices) or excessive when growers adopt conservative overfertilization strategies^8^. Therefore, for improving N management and recommendation guidelines, both soil and plant processes should be considered for defining optimal N fertilization rates and application schedules.

Improving our understanding of the co-regulation of N uptake by the availability of N from the soil and plant N demand is relevant to define the overall critical N plant concentration (%Nc), herein defined as the minimum plant N concentration (%N) required to achieve maximum crop mass (W) for a given range of crop species in different environments and N-fertilized conditions. This critical value %Nc is known to decline over time as W increases, and the relationship between %Nc and W defines a function named “critical N dilution curve”^9^. This function is very useful to conduct crop N diagnosis because it allows the computation of the N nutrition index (NNI), defined as the ratio of the actual %N (%Nact) to the value of %Nc corresponding to the observed value of W (Wact). A NNI value close to 1 indicated N sufficiency, while a NNI below or above 1 reveal N deficiency and excessive N status, respectively.

Critical N dilution curves were established for several major field crops, including wheat (*Triticum aestivum* L.)^10^,winter oilseed rape (*Brassica napus* L.)^11^, maize (*Zea mays* L.)^12^, potato (*Solanum tuberosum* L.)^13^, rice (*Oryza sativa* L.)^14^, and tall fescue (*Festuca arundinacea* Schreb.)^15^, among other crops. These curves have been parametrized using either data collected in single experiments or data set pooled from multi-year and multi-sites experiment networks. These curves are now used by many agricultural scientists and engineers for improving fertilization guidelines based on NNI. Many investigations were recently conducted to develop site-specific critical N dilution curves in different parts of the world for many crop species^10–15^. However, the scientific value of such local critical N dilution curves is debatable because these curves were parametrized using a small number of local data, leading to high uncertainty and to a risk of false discoveries^16^. Although sensitivity analyses were performed in some of these studies, they generally suffer from inadequate protocols and methods^17^. Thus, there is a need for generic and robust critical N dilution curves valid across a large number of crop species in different environments and N-fertilized conditions^18^. To date, any attempt to develop generic critical N dilution curves has been hampered by the lack of a reliable large-scale dataset. Yet, a global dataset including a large number of pairs of W and %N values is required to drive research efforts on N plant status diagnosis.

Here, we present a new dataset resulting from a systematic search of all data made publicly available on critical N dilution curves. Our dataset contains 4454 observed pairs of plant W and %N for 19 major crop species collected between 1982 to 2021 in 16 countries worldwide. These data were extracted from a total of 36 peer-reviewed scientific manuscripts. It offers a unique source of information for developing generic critical N dilution curves and identifying knowledge gaps to guide future research programs on N plant status diagnosis.

## Methods

### Data collection

**Fig. 1:**
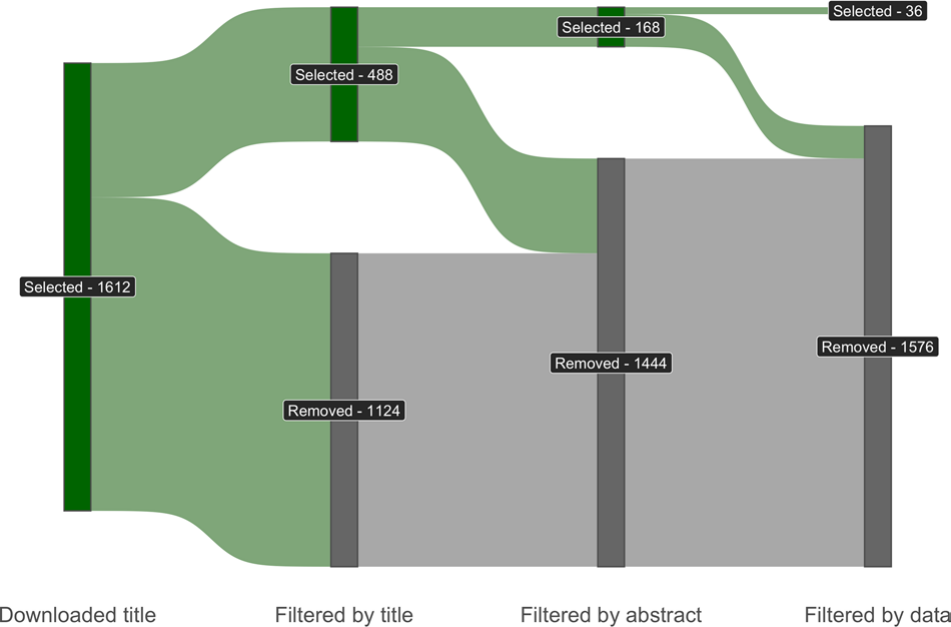
Sankey diagram describing paper search, collection, filtering, and selection.

**Fig. 2:**
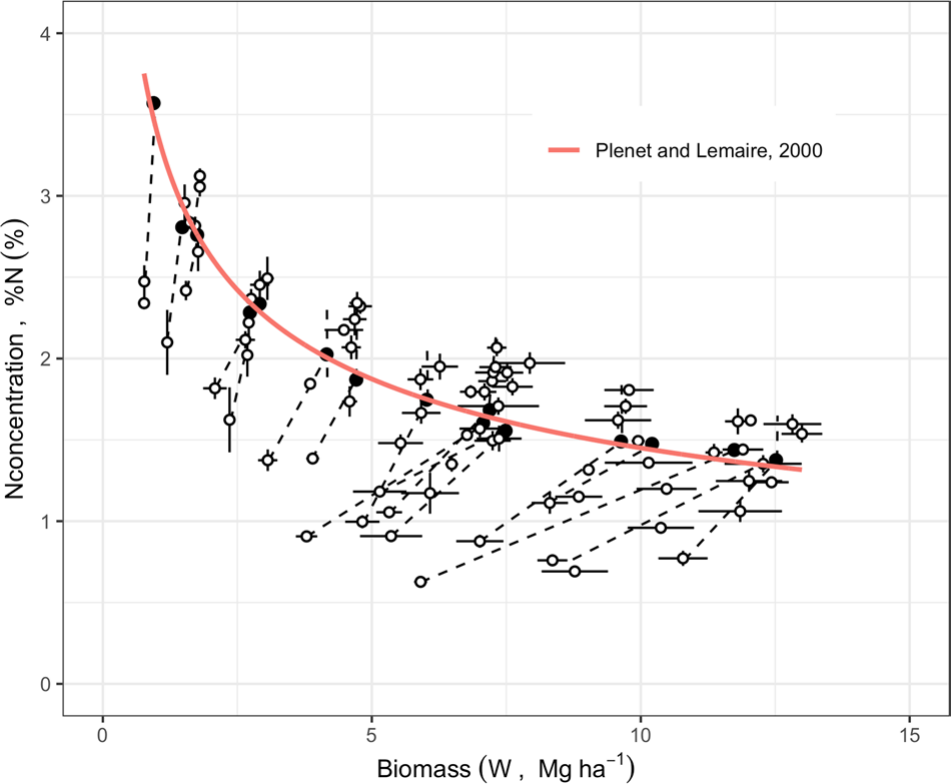
Standard framework considered to determine critical N dilution curve for plant N concentration (%) and plant biomass (W). The white points correspond to observations (bars indicate the standard error of the mean) and the black points correspond to critical %N (minimum %N leading to maximum biomass Wmax) determined by fitting a linear-plus-plateau response model at each sampling time. The critical N dilution curve passes through the black points. Here between 3 and 6 different fertilizer N rates are available at each sampling dates. Data and figure redrawn from Plénet and Lemaire^12^.

**Fig. 3:**
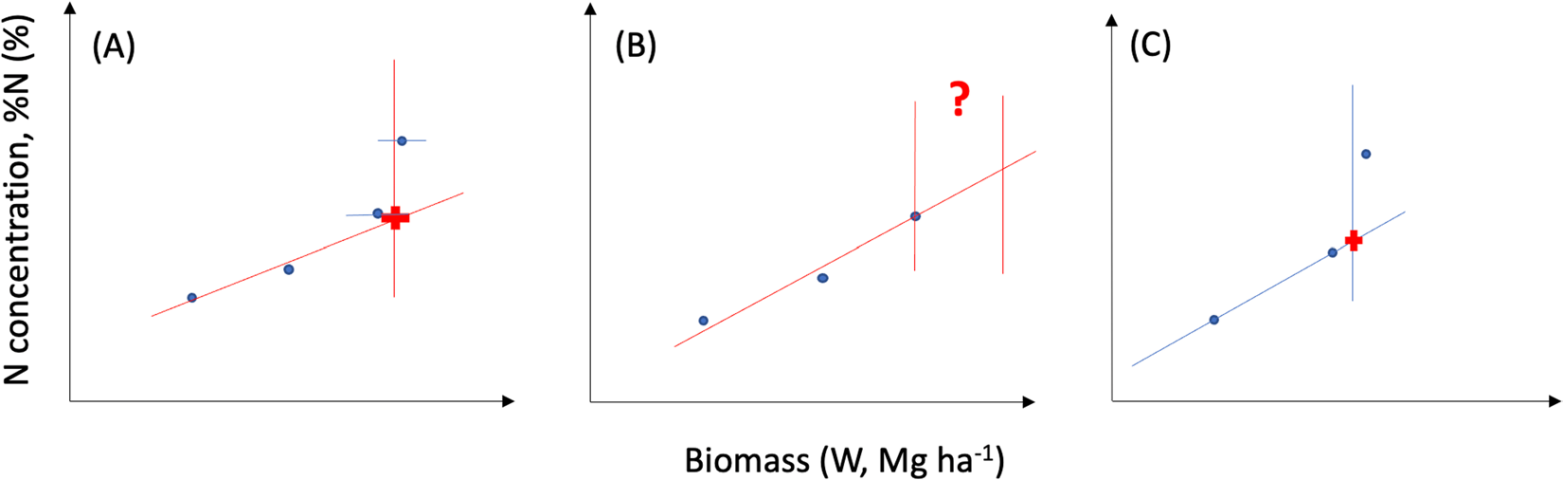
Theoretical representation of the linear-plus-plateau model for the plant N concentration (%N) and plant biomass (W) for three different scenarios (**A**) with an identifiable linear-plus-plateau model with four fertilizer N rates, (**B**) non-identifiable linear-plus-plateau model with three fertilizer N rates (“lack of identifiability”), and lastly (**C**) an identifiable linear-plus-plateau model with three fertilizer N rates.

A literature search was executed between June and August 2021 (2 months) using the following keywords: ‘Nitrogen dilution curve’ & ‘Nitrogen nutrition index’ & ‘Critical nitrogen concentration’ & the name of each crop. The search was performed in the scientific databases ‘ScienceDirect’, ‘Scopus’, and ‘Science Citation Index (Web of Science)’. A last update on this revision to conclude the dataset and include any potential new studies was executed during January 2022. The overall selection process was conducted using the R package *revtools*^19^. A total of 1612 potentially critical papers were identified by screening the title of the paper, and the following steps on the selection process are highlighted in Fig. 1. The first step filtered papers by excluding studies that did not have relevant keywords and abstract information, resulting in the exclusion of 1124 papers. As the second step, manuscripts not reporting data of W and %N for different sampling times (corresponding to different vegetative stages of fields crops) were excluded; a total of 320 papers were removed at this step. At the third step, relevant studies were selected using the following criteria: i) at least 3 or more sampling dates during the vegetative period are recommended to explore a large variation on both plant W and %Nact to achieve a reasonable level of uncertainty for the critical N dilution curve (Fig. 2), ii) reporting of plant W and %Nact, iii) at least 3 or more fertilizer N rate levels, iv) reporting of crop, location, and year of experiment. In each study, three or even more N fertilizer rates are required to discriminate the non-limiting from the limiting N data, and then to determine the maximum plant W (Wmax) achieved at each sampling date. Data were visually inspected to verify that at least one biomass could be considered obtained under non-limiting nitrogen conditions on each observation date. This step helps to assure that from the studies included within a crop, a plateau of plant W has been achieved reducing the uncertainty for the estimation of the critical plant %N in the dilution model. In addition, the determination of critical %N-W data point require the fitting of a linear and plateau %N -W model (Fig. 2). To avoid issue related to lack of identifiability (lack of sufficient data to allow the estimation of the linear-plus-plateau model and the determination of the critical %N-W model), it is necessary to have at least three fertilizer N rate to fit this type of model (Fig. 3). Based on these criteria, 132 papers were discarded (Fig. 1). The total number of papers retained from this search were 36, each paper provided data for one crop except for two papers providing data for two crops each and another one for four crops, totalizing 41 entries (further details presented in Table 1).

**Table 1:** Study identification (ID), author/year, species, country, experimental design, years of study, fertilizer N levels and rates, genotypes, number of observations (and sampling times), and main topics for 19 crop species around the globe.

StudyID	Author/year	Species	Country	Experimental design	Years of study	Fertilizer N levels & rates (kg ha^−1^)	Genotypes	Observations &sampling times	Main topics
1	Agnusdei *et al*. (2010)	Annual ryegrass	Argentina	Split-plot in randomized complete block	1994/1995/1997	6 (0, 50, 100, 150, 200 & 250)	Grasslands Tama	102 (6)	Critical N concentration; N nutrition index; Forage grasses
2	Marino *et al*. (2004)	Annual ryegrass	Argentina	Split-plot	1994/1995	6 (0, 50, 100, 150, 200 & 250)	Grasslands Tama	72 (6)	Critical N dilution; N nutrition index; NUE and its components
3	Liu *et al*.(2021)	Broomcorn millet	China	Split-plot	2019/2020	3 (0, 75 & 150)	86, 111, 184, 230, 235, or 298	144 (4)	Dry matter accumulation; Low-N-tolerance
4	Hou *et al*. (2021)	Cotton	China	Randomized complete block	2018	4 (250, 300, 350 & 400)	Not available	80 (5)	Seed cotton yield; N uptake; N use efficiency; Soil NO_3_-N
5	Chakwizira *et al*. (2016)	Fodder beet	New Zealand	Randomized complete block	2011/2012/2013/2014	4 (0, 50, 100 & 200)	Not available	72 (6)	Allometric relationship; N deficiency; Luxury uptake
6	Sandaña *et al*. (2019)	Hybrid ryegrass	Chile	Split-plot	2016	7 (0, 50, 100, 200, 350, 525 & 700)	Trojan and Shogun	294 (10 and 11)	Forage yield; N concentration; NNI
7	Barbieri *et al*. (2013)	Maize	Argentina	Split-plot in randomized complete block	1996/1999/2000	4 (0, 90, 140 & 180)	DK639 and DK615	86 (4)	Maize; row spacing; N status
8	Chen *et al*. (2013)	Maize	China	Randomized complete block	2011	5 (0, 70, 140, 210 & 280)	Zhengdan 958	40 (8)	Critical N curve; NNI; remote sensing
9	Ciampitti *et al*. (2013)	Maize	United States	Split-split-plot	2010/2011	3 (0, 112 & 224)	2M750 and 2T789	432 (6)	N use efficiency; shoot N remobilization; grain N
10	Li *et al*. (2012)	Maize	China	Randomized complete block	2008/2009	6 (0, 70, 140, 210, 280 & 350)	Zhengdan 958	169 (5)	Critical N concentration; N nutrition index; spring maize
11	Massignam *et al*. (2009)	Maize	Australia	Randomized complete block	1999/2001	Different N rates (0, 20, 50, 70, 150, 250, or 300)	Hycorn53	73 (7, 8 and 9)	Grain yield; Biomass; N uptake; Radiation use efficiency
12	Plénet and Lemaire 2000)	Maize	France	Randomized complete block	1990/1991/1992/1993	Different N rates (30, 50, 80, 100, 120, 130, 180, 240, 280, or 300)	Volga	215 (6, 7 and 12)	Critical N concentration; plant nitrate test, radiation use efficiency; N use efficiency
13	Ranjbar *et al*. (2020)	Maize	Iran	Randomized complete block	2015/2016	7 (0, 50, 100, 150, 200, 250 & 300)	SC 704	84 (6)	Canopy cover; dry matter; N management
14	Texeira *et al*. (2014)	Maize	New Zealand	Randomized complete block	2012/2013	3 (30, 75 & 250)	Pioneer 39G12	30 (5)	Corn; Radiation; N; Sustainability; Water
15	Wen *et al*. (2015)	Maize	China	Split-plot	2012/2013	5 (0, 80, 160, 240 & 320)	Fengtian no. 6	167 (6)	Irrigation regimes; N; yield
16	Zhao *et al*. (2018)	Maize	China	Randomized complete block	2015/2016	Different N rates (0, 75, 90, 150, 180, 225, 270, or 300)	Zhengdan 958 and DH605	90 (5)	Summer maize; Critical N concentration; N nutrition index
17	Zaidi *et al*. (2008)	Maize	Canada	Randomized complete block	2004/2005	Different N rates (20, 50, 73, 100, 125, 150, 178, 200, or 250)	P39W54, P39D82, P38A24 and DKC-4627 BT	144 (4, 5 and 6)	Critical N concentration; N nutrition index; N concentration
18	Agnusdei *et al*. (2010)	Oat	Argentina	Split-plot in randomized complete block	1994/1995	6 (0, 50, 100, 150, 200 & 250)	Grasslands Tama	70 (6)	Critical N concentration; N fertilization; N nutrition index; Forage grasses
19	Salette *et al*. (1982)	Perennial ryegrass	France	Split-plot	1982	3 (0, 120 & 180)	Not available	19 (6)	Dry matter accumulation curve, N uptake; %N curves
20	Belanger *et al*. (2001)	Potato	Canada	Split-plot	1996	4 (0, 50, 100 & 250)	Russet Burbank	44 (5 and 6)	N fertilizer; irrigation; cultivars
21	Trawczyński (2019)	Potato	Poland	Randomized block	2008/2010	5 (0, 50, 100, 150 & 200)	Gwiazda, Etiuda, and Gustaw	45 (3)	Leaf greenness index; N nutritional status
22	Agnusdei *et al*. (2010)	Rescue grass	Argentina	Split-plot in randomized complete block	1997	6 (0, 50, 100, 150, 200 & 250)	Martin Fierro	30 (6)	Critical N concentration; N nutrition index; Forage grasses
23	Ata-UI-Karim *et al*. (2013)	Rice	China	Not available	2010/2011	5 (0, 80, 160, 240 & 320)	Lingxiangyou-18 and Wuxiangjing-14	120 (6)	Critical N dilution curve; Nitrogen nutrition index; Shoot biomass
24	He *et al*. (2017)	Rice	China	Completely randomized block	2013/2014	Different N rates (0, 75, 90, 150, 180, 225, 270, 300, or 360)	Tanliangyou-83, Zhongjiazao-17, Tianyouhuazhan, Yueyou- 9113	218 (5, 6 and 7)	Late rice; early rice; critical N dilution curve; N nutrition index, shoot biomass; yield
25	Yang *et al*. (2014)	Rice	China	Randomized block	2011/2012	5 (0, 75, 150, 225, 300 & 375)	Xiushui63 and Hang43	221 (5)	Grain yield; leaf position; N nutrition index; SPAD values
26	Yang *et al*. (2018)	Rice	China	Randomized block	2010	5 (0, 75, 150, 225, 300 & 375)	Xiushui63	30 (5)	N nutrition index; SPAD values; leaf position; N concentration
27	Yao *et al*. (2021)	Rice	China	Randomized block	2018/2019	5 (0, 75, 150, 225 & 300)	Huiliangyou 898,Y Liangyou 900	120 (6)	Critical N concentration; N nutrition index
28	Cosentino *et al*. (2012)	Sorghum	Italy	Split-plot	1999	4 (0, 60,120 & 180)	Keller	95 (8)	Sweet sorghum; biomass; water balance; critical N dilution curve
29	De Oliveira *et al*. (2013)	Sugarcane(first season and ratoon)	Brazil	Split-plot under a complete randomized block	2005/2006/2007	Different N rates (0, 40, 50, 80, 100, 120 or 150)	SP81-3250	112 (4, 5 and 6)	N fertilization; critical N level; N nutrition index; Saccharum spp.
30	Massignam *et al*. (2009)	Sunflower	Australia	Randomized complete block	1999/2001	Different N rates (0, 20, 50, 70, 150, 250, or 300)	Hysun 36	67 (7 and 8)	Grain yield; Biomass; N uptake; Radiation use efficiency
31	Lv, Zunfu *et al*. (2020)	Sweet potato	China	Randomized block	2018/2019	5 (0, 45, 90, 135 & 180)	Xinxiang and Shang19	120 (6)	Critical N concentration; N nutrition index
32	Agnusdei *et al*. (2010)	Tall fescue	Argentina	Split-plot in randomized complete block	1996	5 (0, 50, 100, 150, 200 & 250)	El Palenque and Maris Kasba	39 (4)	Critical N concentration; N nutrition index; Forage grasses
33	Errecat *et al*. (2014)	Tall fescue	Argentina	Split-plot	2008/2009/2009	Different N rates (0, 75, 150, 225 350 & 500)	El Palenque MAG INTA	195 (6)	Critical N concentration; Water availability.evapotranspiration
34	Lemaire and Denoix (1987)	Tall fescue	France	Not available	1977	4 (0, 50, 100 & 150)	Ludelle	28 (7)	Growth curves; water consumption; evapotranspiration
35	Gastal and Lemaire (1988)	Tall fescue	France	Split-plot	1987	Different N rates (0, 40, 50, 80, 100, 120, 150, or 160)	Clarine	44 (4 and 5)	Growth curves, N uptake and plant %N curves.
36	Salette *et al*. (1982)	Tall fescue	France	Split-plot	1979	Different N rates (50, 60, 100, 120, 150, or 180)	Ludelle	40 (7)	Growth curves, N uptake and plant %N curves
37	Bélanger and Ziadi (2008)	Timothy grass	Canada	Split-split-plot	1999/2000/2001/2002	4 (0, 60, 120 & 180)	Champ	256 (4)	Critical N and P concentrations; Critical N concentration
38	Guo *et al*. (2020)	Wheat	China	Split-plot	2016	5 (0, 90, 180, 270 & 360)	Zhoumai 27	70 (7)	Water conditions; critical N concentration; N nutrition index
39	Justes *et al*. (1994)	Wheat	France	Randomized complete block	1985	4 (80, 123, 166 & 210)	Fidel	24 (6)	N concentration; biomass; critical N dilution
40	Ziadi *et al*. (2010)	Wheat	Canada	Randomized complete block	2004/2005/2006	6 (0, 40, 80, 120, 160 & 200)	AC Barrie	140 (3, 4, 5 and 7)	Critical N dilution curve; N nutrition index
41	Ekbladh and Witter (2010)	White cabbage	Sweden	Not available	2001	4 (0, 100, 225 & 375)	Heckla	13 (3)	Growth rate; Leaf area; N use efficiency

**Fig. 4:**
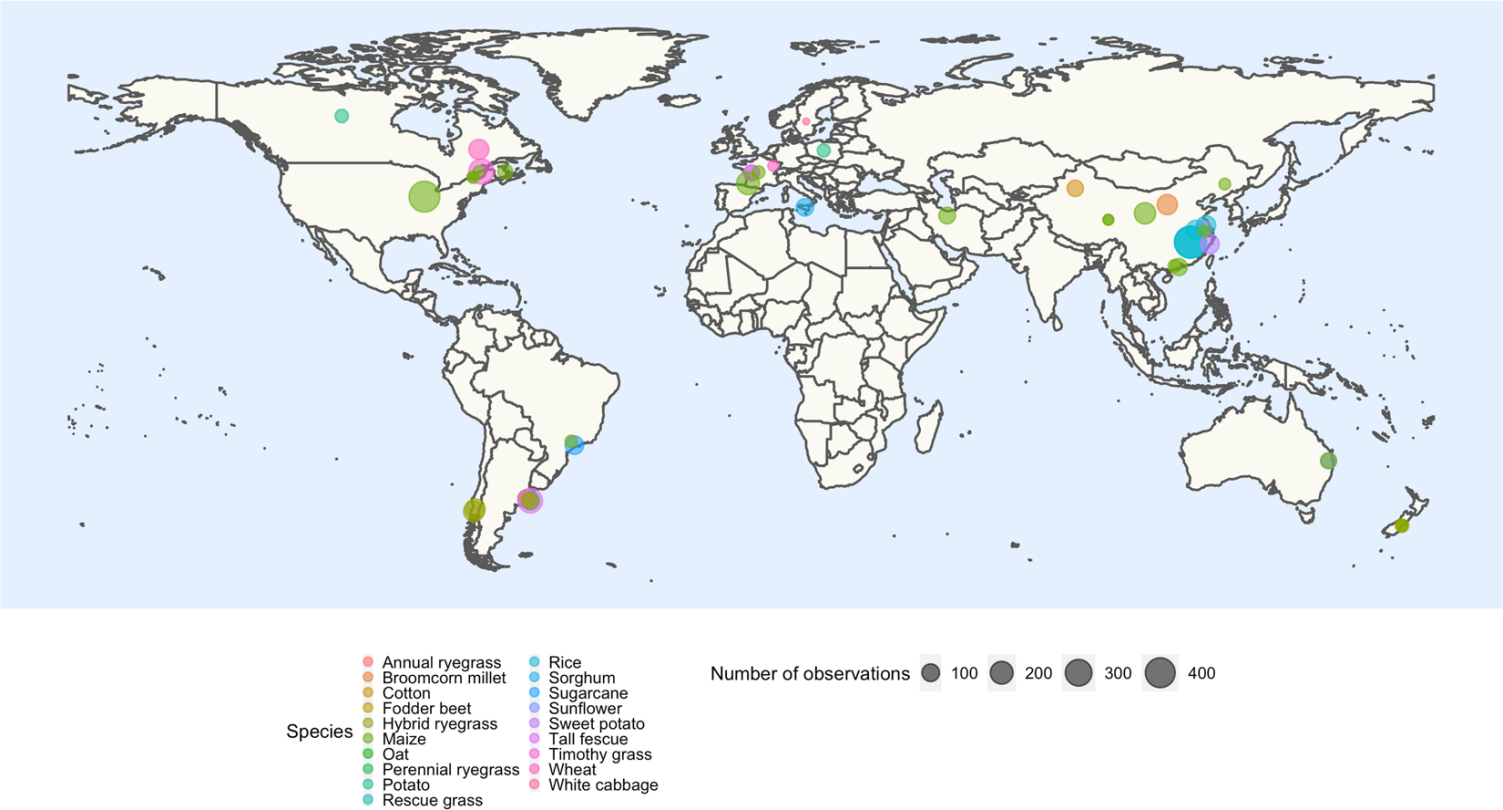
Geographical distribution of the observations included in the dataset. The distinct sizes of the circles indicate the amount of data (one observation corresponds to a pair of crop biomass and plant N concentration) for a given species at a given location, while point colors indicate crop species.

The data from all those papers were manually extracted (with the main plant traits reported or derived), together with relevant details from each study (e.g., author, year of experimentation, fertilizer N rates, description of treatments). If the data were not available in table format, information was retrieved from figures. Data extraction was assisted using the R package juicr^20^. The main crop species, papers, number of observations and geographical distribution of the data are presented in Fig. 3. Among the 36 papers selected (between 1982 and 2021), 33 report data for one crop species, 2 papers report data on 2 crops, and one study provides data on 4 crops. The dataset includes 4454 pairs of observed W and %N for different field crops and N fertilizer rates (Table 1). For each of the 19 crop species reported on this study, the total number of pairs of observed W and %N per crop was: annual ryegrass (*Lolium multiflorum* Lam.) 174, broomcorn millet (*Panicum Miliaceum* L.) 144, cotton (*Gossypium hirsutum* L.) 80, fodder beet (*Beta vulgaris* L. ssp. *vulgaris*) 72, hybrid ryegrass 294, maize 1530, oat (*Avena sativa* L.) 70, perennial ryegrass (*Lolium perenne* L.) 19, potato 89, rescue grass (*Bromus catharticus* H.B.K.) 30, rice 709, sorghum (*Sorghum bicolor* L.) 95, sugarcane (*Saccharum officinarum*) 112, sunflower (*Helianthus annuus* L.) 67, sweet potato *(Ipomoea batatas)* 120, tall fescue 346, timothy grass (*Phleum pratense* L.) 256, wheat 234 and white cabbage (*Brassica oleracea* L.) 13 observations in 16 countries (Fig. 4).

## Data Records

The data are accessible on the figshare repository^21^, available at 10.6084/m9.figshare.19105049.v1, and which includes the following files:

1. “NNI_Database.csv” includes the data.
2. “Summary of the database.docx”, includes a summary of the dataset (meta-data), defining each column, trait collected in the data and the units for each variable.
3. “List of references.docx”, presents all the references of the publications included in the dataset.
4. “Figures_NNI.zip”, includes all the codes to build the figures of this study.

The “NNI_Database” contains all the information collected on this systematic analysis. The “Summary of the database” presents a description of the “NNI_Database” file with the information separated into three categories:

Category I, general details about the dataset, comprising information for author and publication year, and DOI or other identification for each study included in the dataset.

Category II, relevant to the study, defining species, country, experimental design, years of study, fertilizer N rates levels (and rates, kg ha^−1^), crop material (varieties/hybrids).

Category III, key for the dataset related to the pairs of crop biomass (W) and plant N concentration (%N), number of observations per identification number (ID). All the information of W and %N are reported in dry matter basis, as expressed in the data collected from those respective studies.

Table 1 describes the main characteristics of the 41 selected studies, including a specific identification number for each study by crop (ID, from 1 to 41 total), species, country for the study location, author, experimental design of the field trial, years where the study was carried out, number and N fertilizer rates, information on crop material, number of total observations, and relevant keyword for the study.

**Fig. 5:**
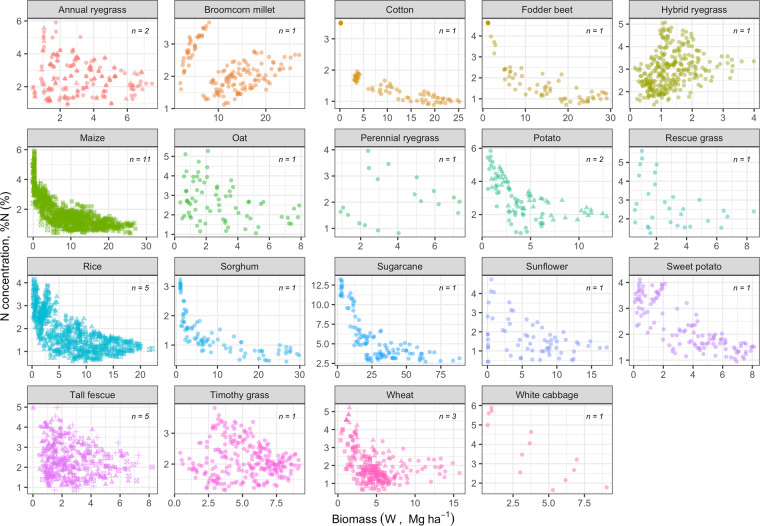
Relationship between plant N concentration (%N) and crop biomass (W) for 19 different crop species (annual ryegrass, broomcorn millet, cotton, fodder beet, hybrid ryegrass, maize, oat, perennial ryegrass, potato, rescue grass, rice, sorghum, sugarcane, sunflower, sweet potato, tall fescue, timothy grass, wheat, and white cabbage). Colors represent different crop species, and *n* represents the number of studies for each species.

Data of plant %N and W are presented in Fig. 4 for all 19 species across sampling times starting at early vegetative growth stages. The W-%N relationships presented for different crops reveal contrasted plant N status (Fig. 5).

Observed values of W and %N are typically used to fit critical N dilution curves. Fitted curves can help to delineate situations of luxury (excess of N), sufficiency, and deficient (lack of N) plant N status. A recent review of critical N curves obtained for maize crop^18^ reported negligible differences across studies. This result revealed that it is more relevant to fit generic critical N dilution curves from a large set of studies covering different environments rather than fitting individual curves to local data. Below, we show that our dataset can be used to establish more universal critical N dilution curve than those generally parametrized from local data.

## Technical Validation

To demonstrate the practical value of the dataset, data collected can be used to parametrize a generic critical N dilution curve for the given field crop. After the step on checking for outliers, the field crop W and %N data is used to fit a critical N dilution curve using the Bayesian modeling approach proposed by Makowski *et al*.^22^. The fitted curve for the field crop can be then compared with a reference curve available in the scientific literature. Lastly as the final part of the second step, an independent dataset can be utilized to assess the plausibility of the NNI values derived from the fitted curve compared to the NNI values derived from the reference critical N dilution model established in the literature. Details of all steps are provided below.

### Step 1 - Technical validation of the range of biomass and N%

This first step is relevant for inspecting the data and detecting potential outliers. Errors of data extraction were eliminated by comparing the extracted data with tables and figures of the original manuscripts. For each crop and paper, the data were inspected for outliers (by study and sampling time) based on the interquartile range (IQR) rule detection method^23^ and with boxplots, as summarized in the Supplementary material. Any observation with W or %N beyond the threshold of 1.5 difference for the IQR to third quartile was pinpointed as a potential outlier for each trait documented for those studies (Fig. 5).

**Fig. 6:**
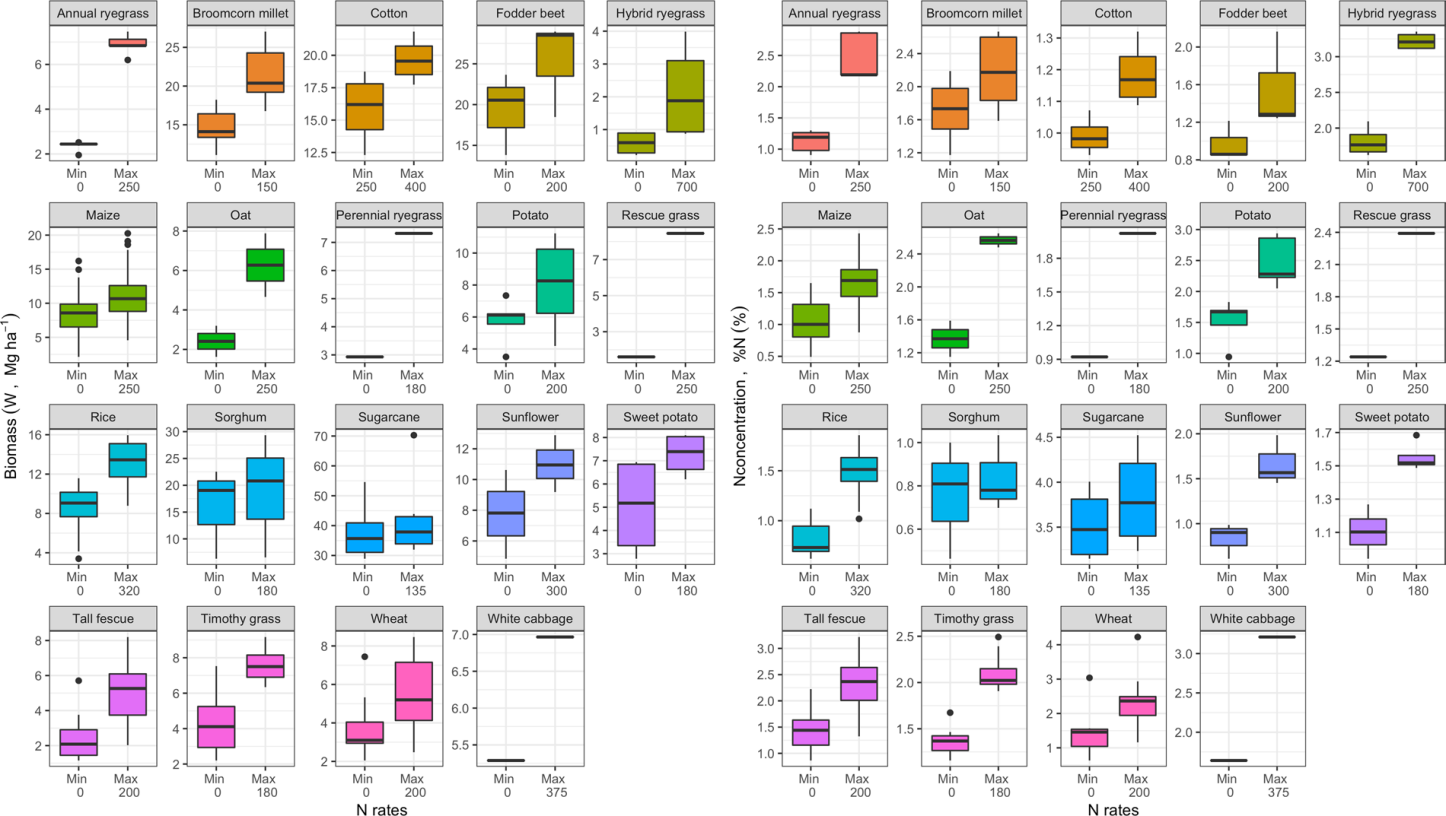
Boxplots for plant biomass (W) and N concentration (%N) for each species for the minimum (Min) and the maximum (Max) fertilizer N rates (median values included in each boxplot for all crop species) utilized in each study. The difference between Max and Min fertilizer N rates defines the N responsiveness of each plant trait (W and %N) for each species. The dots presented in each boxplot refer to the detected outliers based on the interquartile range (IQR) rule detection method^21^.

Lastly, an overall N responsiveness (i.e., the difference between minimum and maximum fertilizer N rate reported by each study within a crop) was calculated to portray the variation on both W and %N obtained from this dataset due of differential N availability (Fig. 6). The N responsiveness was calculated as the difference between the medians of the maximum minus minimum fertilizer N rates across studies within a crop species [((median for maximum rate – median for minimum rate)/(median for minimum rate)) × 100]. Species were then ranked according to their responsiveness to N fertilizer for W and %N, respectively. For W, the species order from high to low responsiveness was: rescue grass (431%), hybrid ryegrass (217%), annual ryegrass (183%), oat (163%), perennial ryegrass (152%), tall grass (152%), timothy grass (83%), wheat (68%), maize (49%), rice (47%), broomcorn millet (45%), sunflower (40%), fodder beet (39%), white cabbage (32%), sweet potato (42%), potato (36%), cotton (21%), sorghum (9%) and sugarcane (6%). The ranking was similar for %N, with grasses portraying the largest differences near 100% between N rates. In contrast, negligible differences between N rates were observed for sorghum.

### Step 2- Fitting a critical N dilution curve for maize

**Fig. 7:**
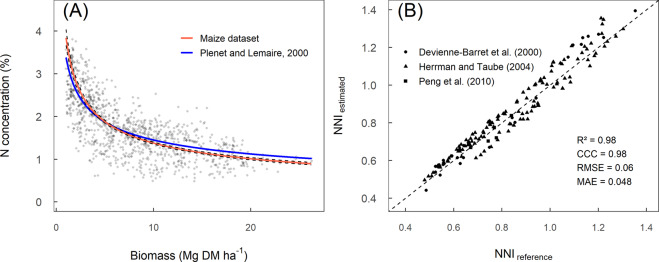
Validation of a critical N dilution curve for maize field crop estimated using the current dataset. (**A**) Blue line represents the reference N dilution curve for maize defined by Plénet and Lemaire:^12^ %Nc = 3.4 *W*^−037^. Red and dashed lines represent the critical N curves (median) and their 95% credible intervals (CI), respectively: %Nc [95%CI] = 3.86 [3.68,4.06] *W*^−0.44 [0.42,0.47]^. (**B**) Comparison between the NNI computed using the reference curve by Plénet and Lemaire^12^ and the critical N dilution curve based on this dataset. Symbols represent the four independent studies (i.e. not included in the main dataset) used for comparison of the NNI estimates. The metrics determined using the metrica package^27^ were concordance correlation coefficient (**C**), relative root mean square error (RMSE), and mean absolute error (MAE).

For this step, a case study was established for maize field crop utilizing a subset of the plant W-%N data from the 11 studies on this crop analyzed using a Bayesian model for estimating coefficients of the critical N dilution curve (Fig. 7). A standard equation was used to relate %Nc to W, specifically %*N*_*c*_ = *A*1 × *W*^−*A*2^, where *A1* and *A2* are two parameters^9–12,18,22^. The modeling and validation of the critical N dilution curve were performed into four phases:

(i) A pre-processing of the data was conducted following the approach used by Plénet and Lemaire^12^ for maize crop. First, the data were filtered to include plant W above 1 Mg ha^−1^. In addition, dates of measures were selected to include maize vegetative and reproductive growth until silking plus 25 days (or approximately until milk stage, R3 growth stage).
(ii) A Bayesian hierarchical model was fitted to the data following the procedure defined by Makowski *et al*.^22^. A Markov chain Monte Carlo algorithm (MCMC) was implemented using the R package *rjags*^24^. The algorithm was first run with three chains of 50,000 iterations each. Convergence was achieved approximately near 50,000 iterations according to visual inspection of the trace plots and Gelman-Rubin diagnosis. These first 50,000 samples were discarded as “burn-in”, and the algorithm was run again during 100,000 additional iterations to determine the posterior medians and 95% credible intervals of *A1* and *A2* for maize crop.
(iii) The results obtained were used to compute the posterior distribution for the critical N dilution curve. The critical N dilution for maize crop was estimated from 1 Mg ha^−1^ to the maximum value of *W* in the dataset. Median and 95% credible intervals were determined and plotted against the reference curve established for maize crop by Plénet and Lemaire^12^ (Fig. 7A).
(iv) The critical N% determined using the Bayesian procedure on this data was compared against the corresponding values estimated by the reference curve for maize^12^. This reference N dilution curve was derived from five studies located in France. To avoid any bias due to the use of the same data for fitting and testing, an independent dataset of four maize studies^25–27^ was used to compute NNI using the two fitted critical N dilution curves (Bayesian N curve fitted to our global dataset vs. the traditional model for maize^12^). Error metrics describing the agreement between NNI values were computed using the *metrica* package^28^. The root mean square error (RMSE) and mean absolute error (MAE) are standard measures of prediction accuracy, quantifying the average magnitude of the errors in the predictions. The concordance correlation coefficient (CCC) is a measure of both accuracy and precision of the model, quantifying the agreement between an estimated and a reference value. From these metrics, the CCC reflected a strong agreement between both models (CCC = 0.96) and both the RMSE (RMSE = 0.053) and MAE (MAE = 0.046) confirmed that the critical N dilution curve developed with this dataset is very similar to the well-established reference curve^12^ available for maize (Fig. 7B). Lastly, the lack of departure of the two critical N dilution curves from the 1:1 line also confirm that these two models derived in similar NNI values. Thus, these critical curves are not statistically different, confirming the past findings for maize crop from Ciampitti *et al*.^18^. Likewise, Fernandez *et al*.^15^ reported a universal critical N dilution curve for 14 environments and N-fertilized conditions for tall fescue. A similar method could be used to fit critical N dilution curves for other species from our data.

## Usage Notes

The current data set can be used to develop critical N dilution curves for the diagnostic of N nutritional status of wide range of crop species in different environments and N-fertilized conditions. Specifically, our dataset can be used to fit critical N dilution curves, assess their uncertainties, and determine the statistical significance of the difference between two critical N dilution curves.

**Fig. 8:**
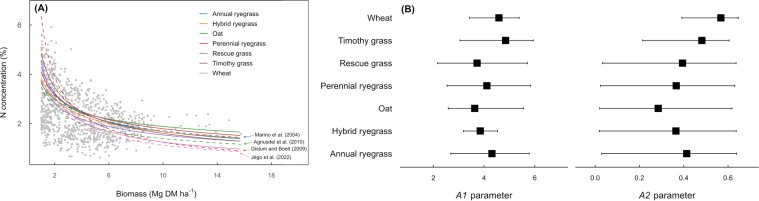
Plant N concentration and biomass for grass species (annual, hybrid and perennial ryegrass, oat, rescue grass, timothy grass, and wheat crop), portraying the critical N dilution curves for grass forages and wheat (Marino *et al*.^29^; Agnusdei *et al*.^30^; Gislum and Boelt^31^; Jégo *et al*.^32^) (panel A), and parameters of the N dilution model (A1, A2), estimates (black squares) and their 95% credibility intervals for each grass species (panel B).

In addition, the current data set could be used for testing the universality of critical N dilution curves among crop species. For example, when comparing the N dilution curves of multiple grasses (annual, hybrid and perennial ryegrass, oat, rescue grass, timothy grass, and wheat crop), we found that the parameters of the model (A1 and A2) did not differ significantly (Fig. 8). For the different species, the N dilution models were benchmarked with the critical N dilution curves established in previous studies. Results show that the curves were relatively similar for all species considered. Results also reveal the uncertainty (reflected as the length of the 95% credibility interval) is higher for the A2 parameter than for the A1 parameter, except wheat crop. The level of uncertainty depends on the number of observations within a study and on the total number of studies for a crop. When the number of data is small, the determination of the critical N curve can produce estimates with large uncertainty (wide credibility intervals).

In the future, our dataset could be easily updated with data generated by new studies and, also, with previous studies where data were not available. Studies to be incorporated in this global dataset may be sent to the corresponding author (IAC, ciampitti@ksu.edu). The goal of our global initiative is expand the dataset by including more data and involve more collaborators. The ambition is the make the largest possible amount of W and %N data available and stimulate the development of reliable critical N dilution curves. In addition to %N, this database could be expanded in near future to cover other nutrients such as phosphorus, sulfur, and potassium, and their interaction with other environmental factors such water stress. Such a collaborative approach may set a milestone in plant physiology and nutrition from which more universal and reliable models could be developed to improve fertilizer management practices and reduce their environmental footprint.

## Supplementary information

Supplementary Information

## Data Availability

Scripts using R programming language are provided to produce figures. Additional code and related files are available at figshare repository^21^: 10.6084/m9.figshare.19105049.v1.
