# Supplementary material for: A global dataset to parametrize critical nitrogen dilution curves for major crop species
Source: Sci Data. 2022 Jun 7;9:277. doi: 10.1038/s41597-022-01395-2 (PMC9174182; doi:10.1038/s41597-022-01395-2)

**A global dataset for nitrogen nutrition index (NNI) for field crops**

**Supplementary File**

Ignacio A. Ciampitti1*, Emmanuela van Versendaal1, Juan Francisco Rybecky1, Josefina Lacasa1, Javier Fernandez1, David Makowski2, and Gilles Lemaire3

1 Department of Agronomy, Kansas State University, Manhattan, Kansas, US
2 University Paris-Saclay, INRAE, AgroParisTech, UMR 518, 75231 Paris, France
3 Honorary Director of Research, INRAE, 86600 Lusignan, France

Table of Contents

[1. Annual ryegrass 3](#_Toc97911639)

[2. Broomcorn millet 4](#_Toc97911640)

[3. Cotton 4](#_Toc97911641)

[4. Fodder beet 5](#_Toc97911642)

[5. Hybrid ryegrass 6](#_Toc97911643)

[6. Maize 7](#_Toc97911644)

[7. Oat 8](#_Toc97911645)

[8. Perennial ryegrass 8](#_Toc97911646)

[9. Potato 9](#_Toc97911647)

[10. Rescue grass 9](#_Toc97911648)

[11. Rice 10](#_Toc97911649)

[12. Sorghum 11](#_Toc97911650)

[13. Sugarcane 12](#_Toc97911651)

[14. Sunflower 12](#_Toc97911652)

[15. Sweet potato 13](#_Toc97911653)

[16. Tall fescue 14](#_Toc97911654)

[17. Timothy grass 15](#_Toc97911655)

[18. Wheat 16](#_Toc97911656)

[19. White cabbage 17](#_Toc97911657)

# Annual ryegrass


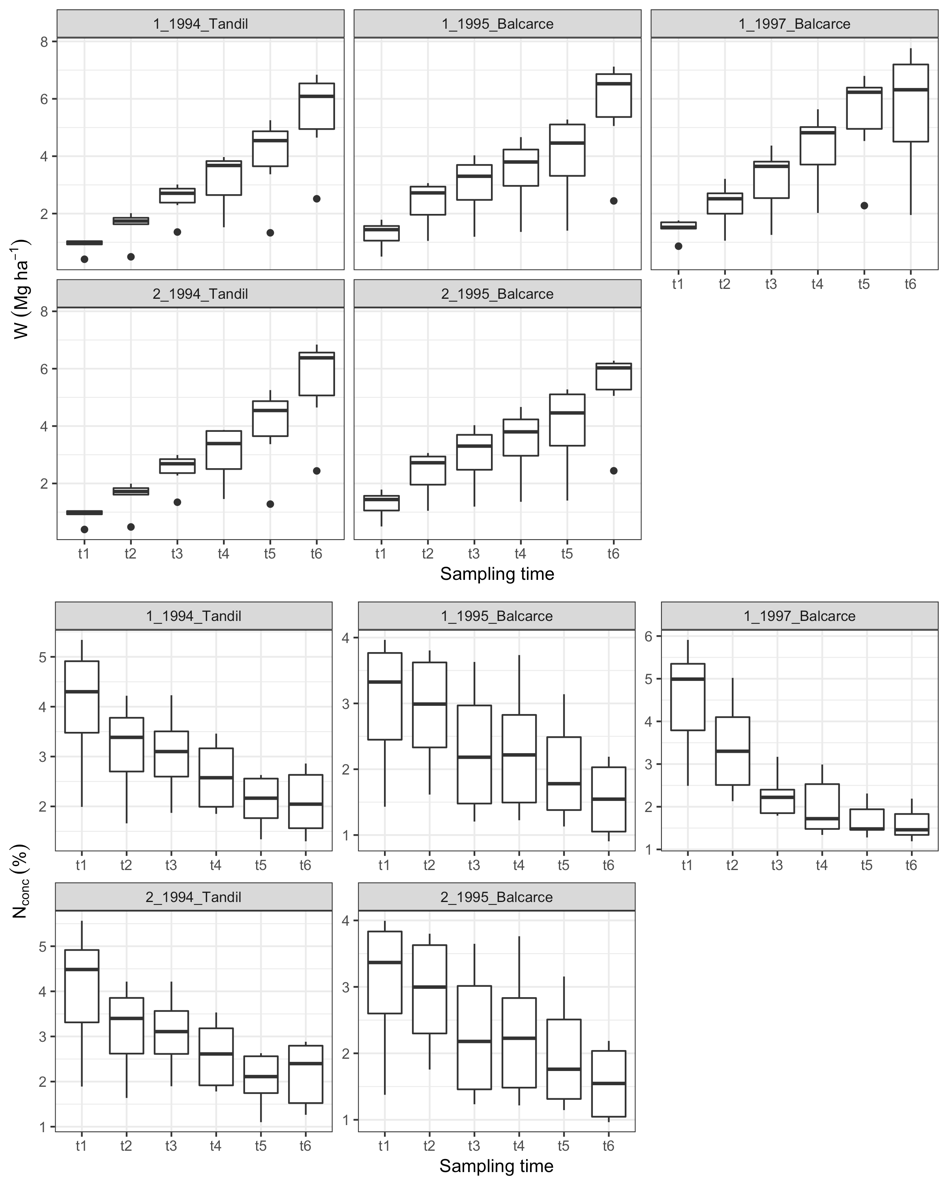


# Broomcorn millet


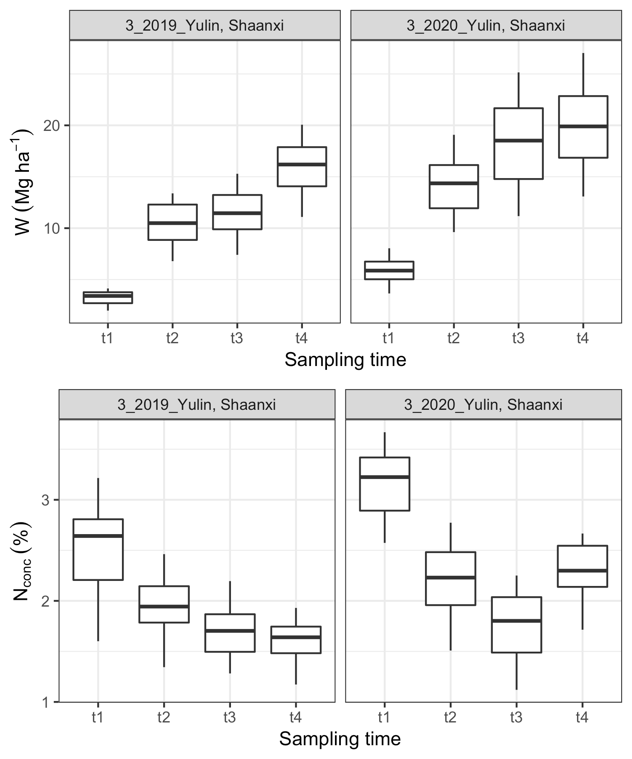


# Cotton


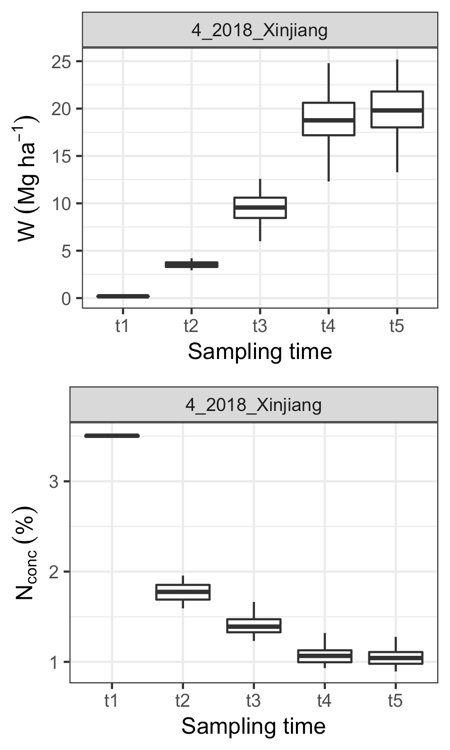


# Fodder beet


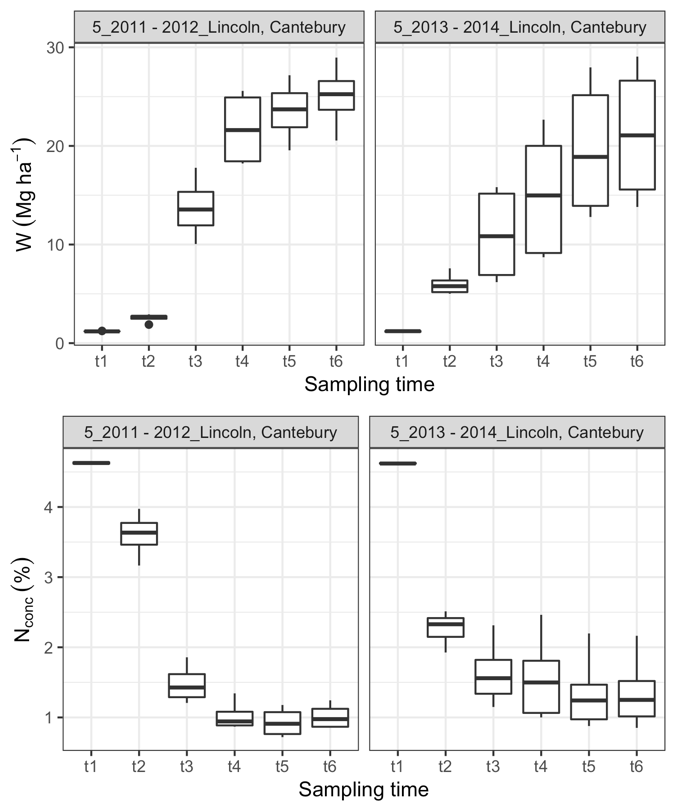


# Hybrid ryegrass


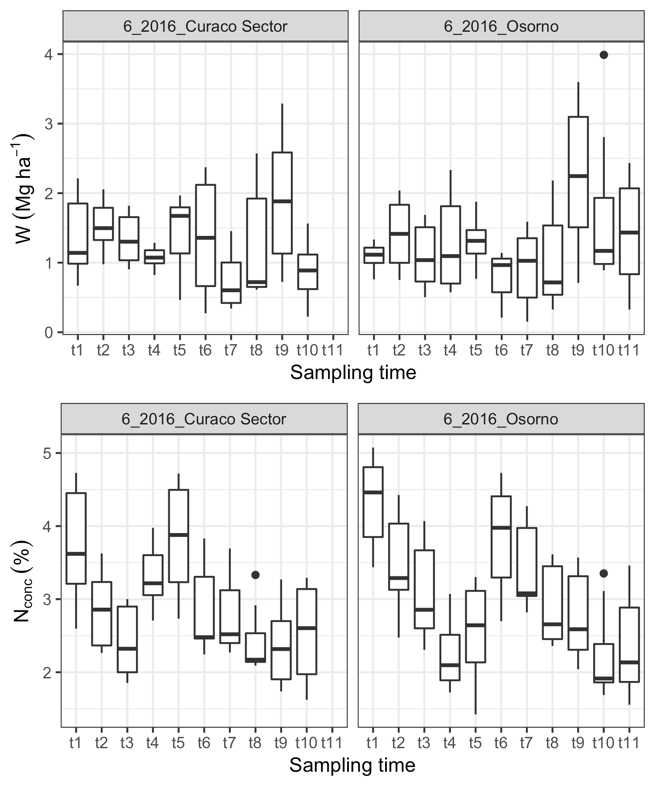


# Maize


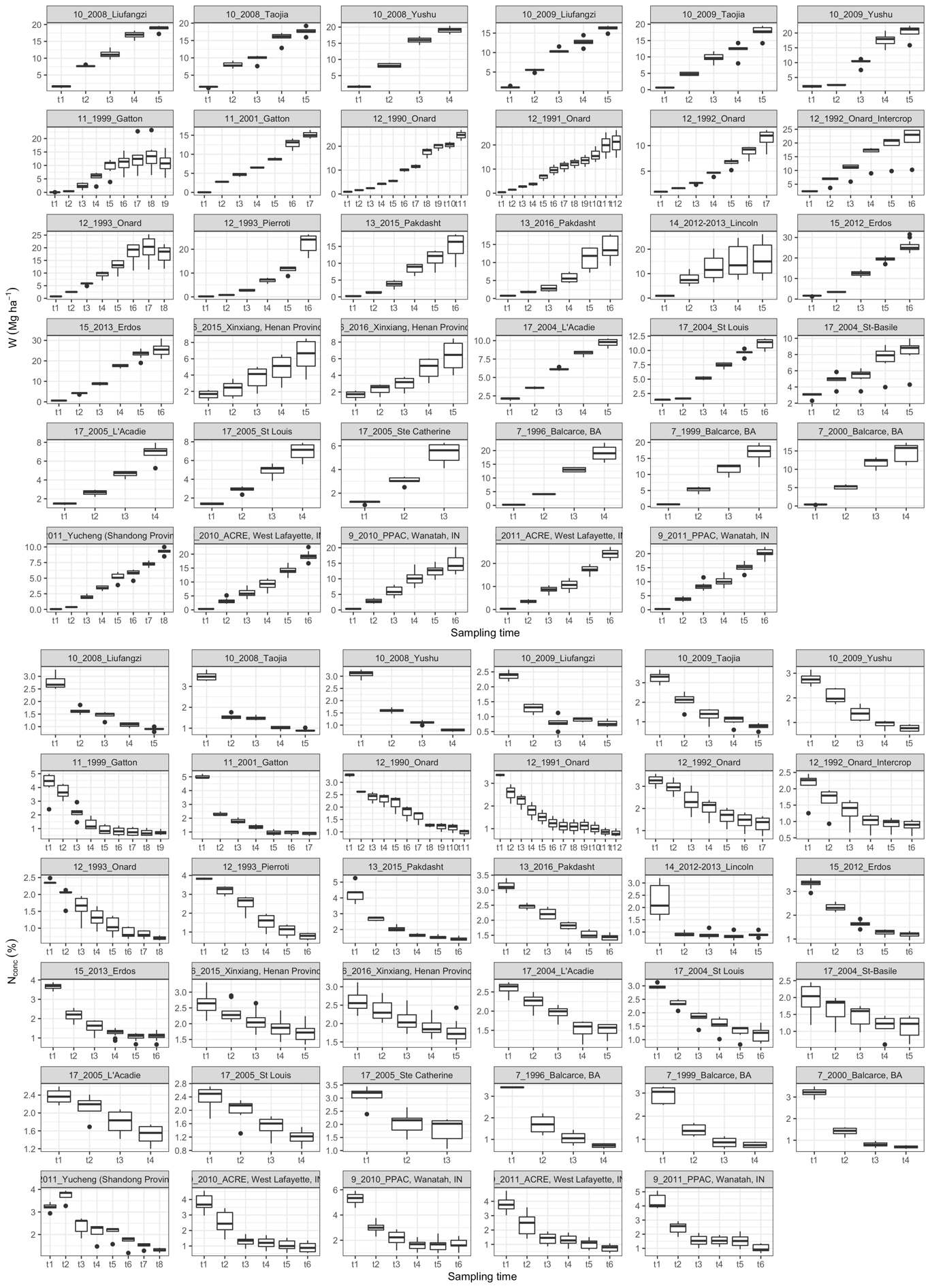


# Oat


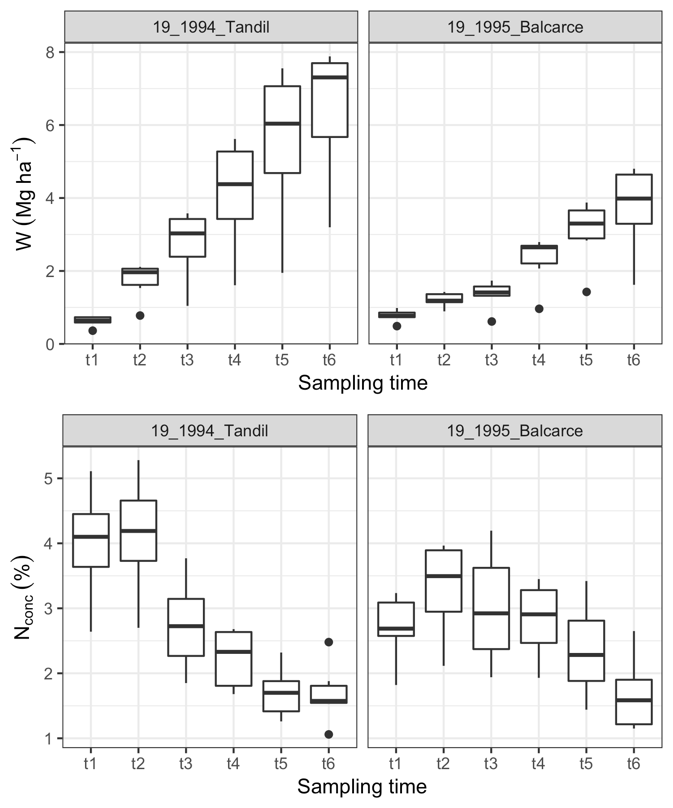


# Perennial ryegrass


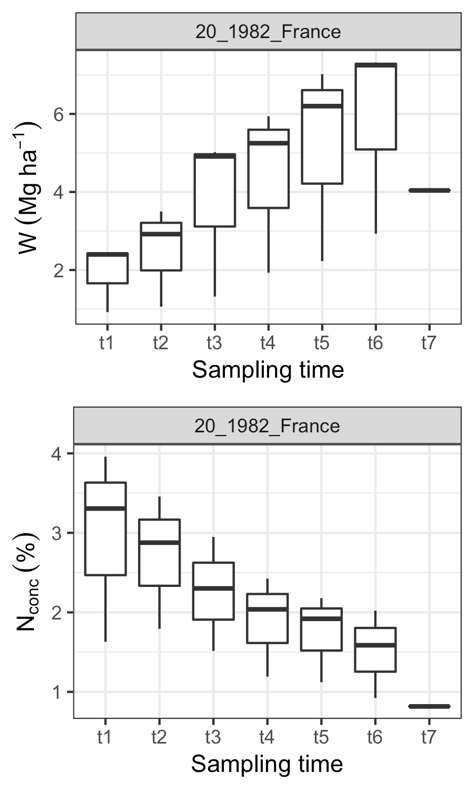


# Potato


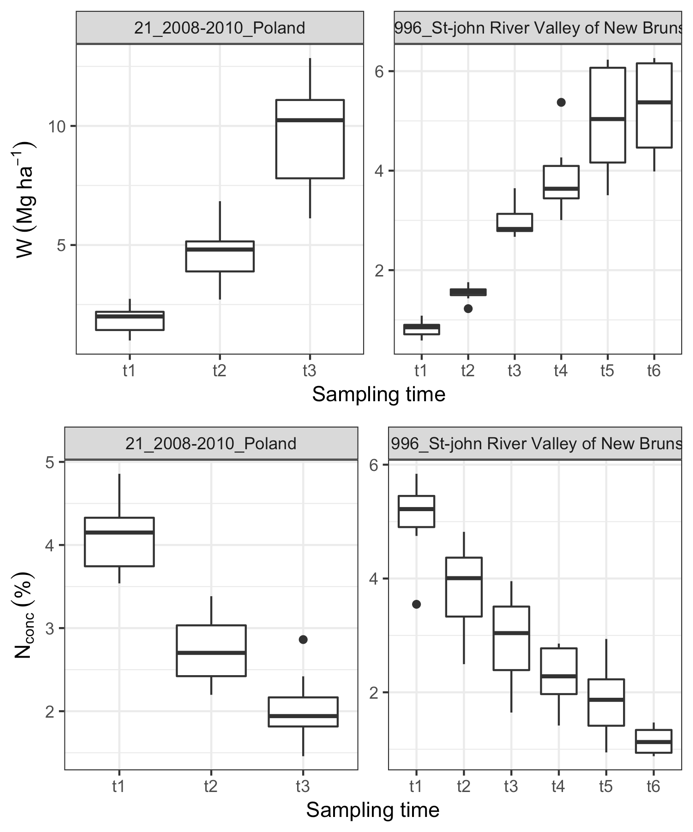


# Rescue grass


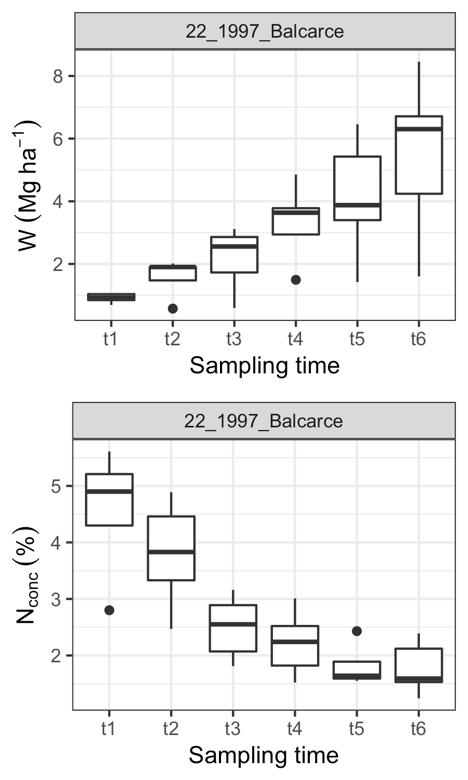


# Rice


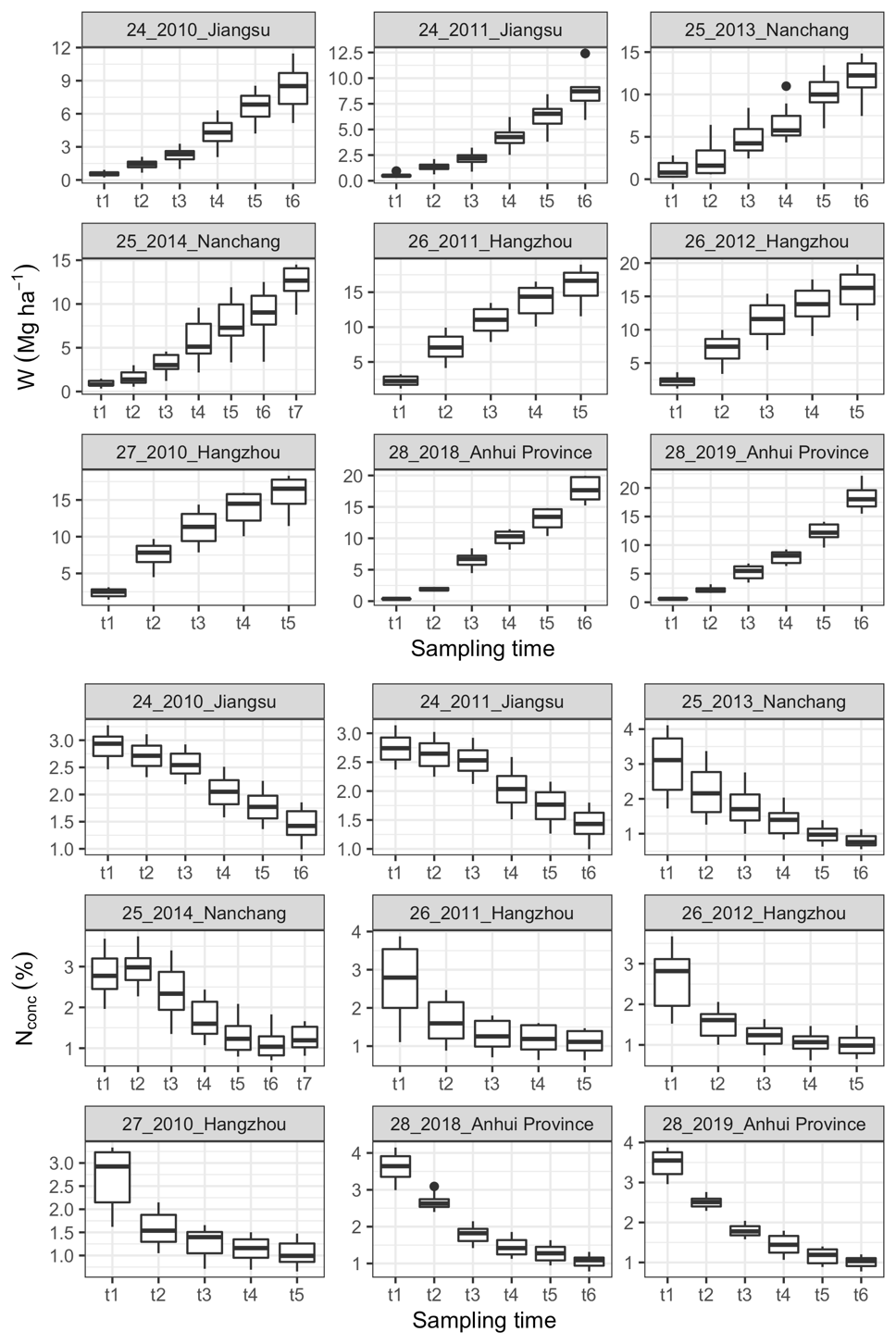


# Sorghum


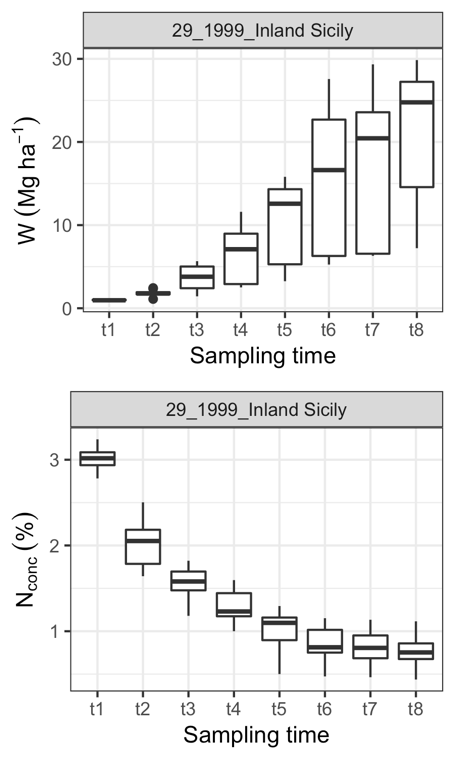


# Sugarcane


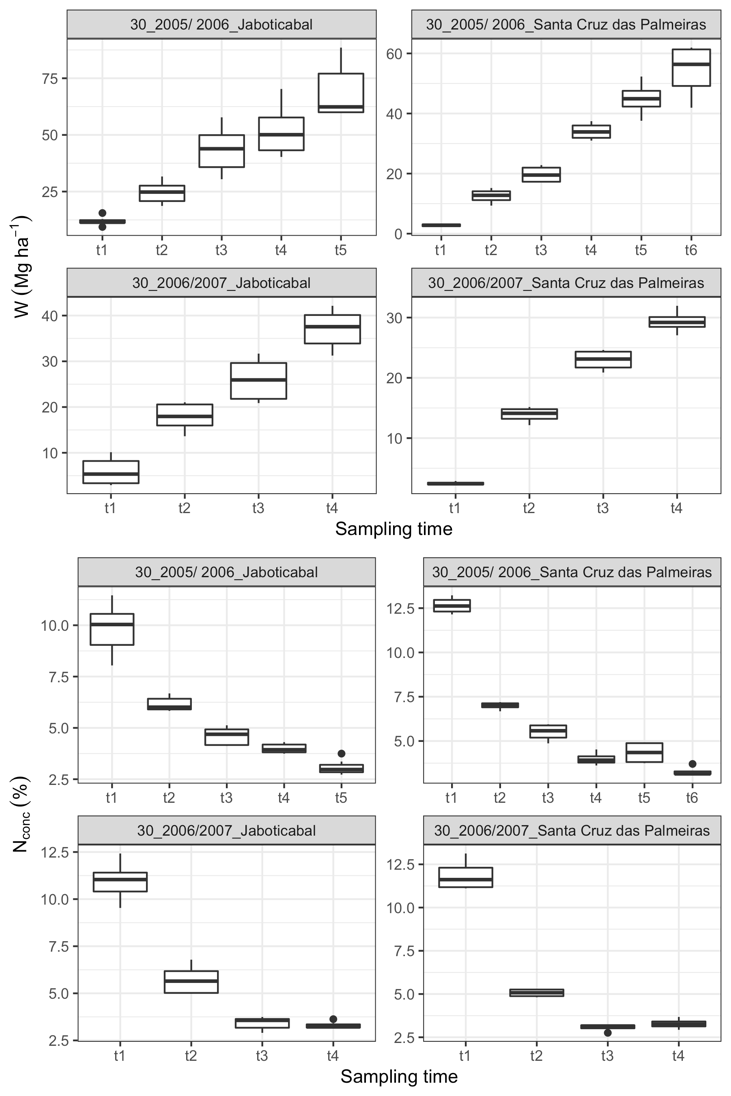


# Sunflower


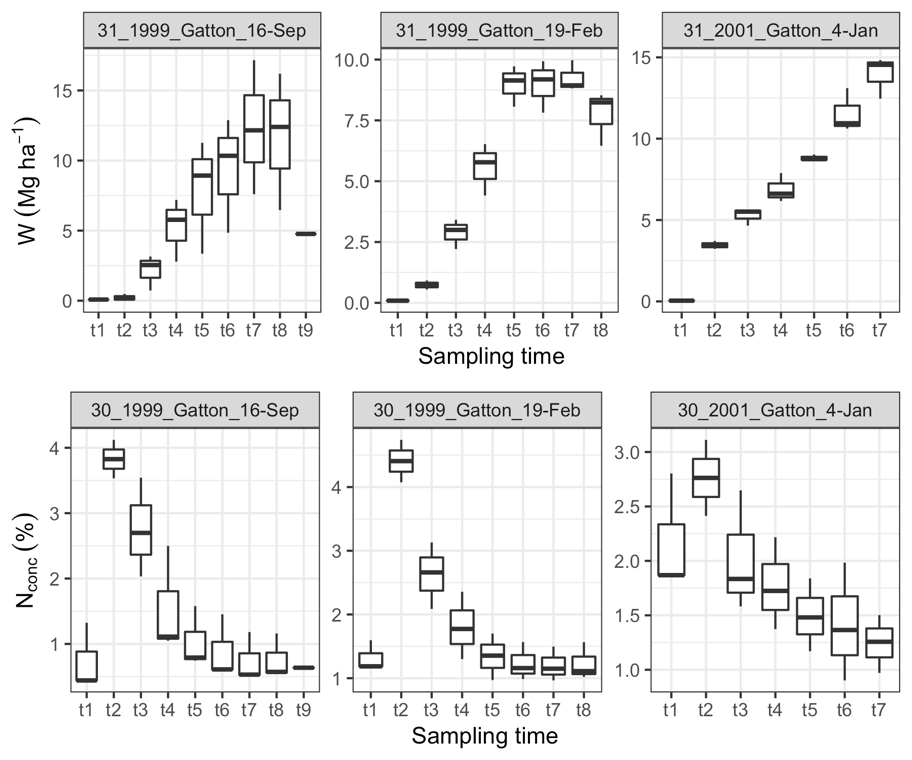


# Sweet potato


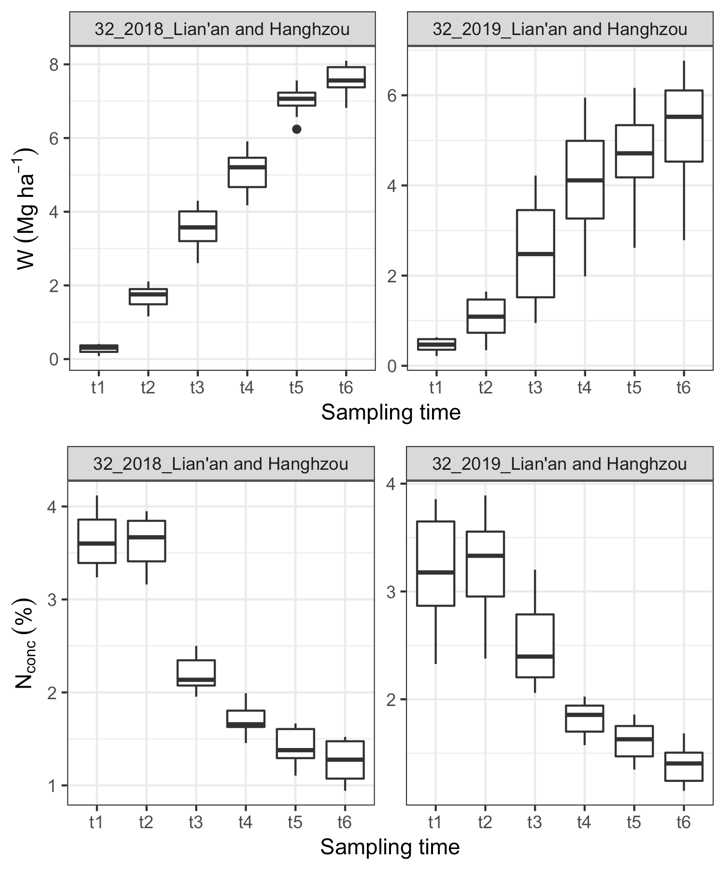


# Tall fescue


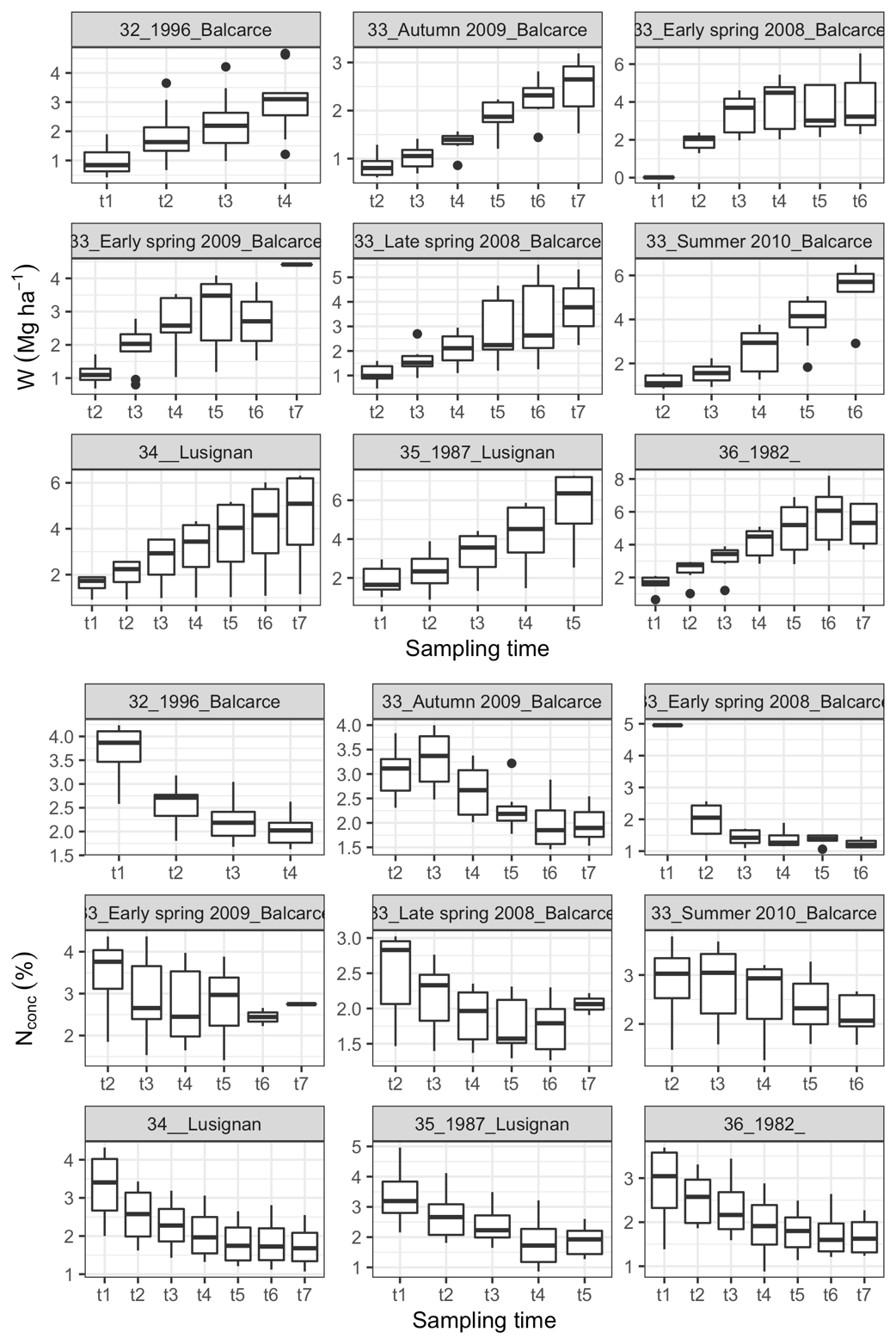


# Timothy grass


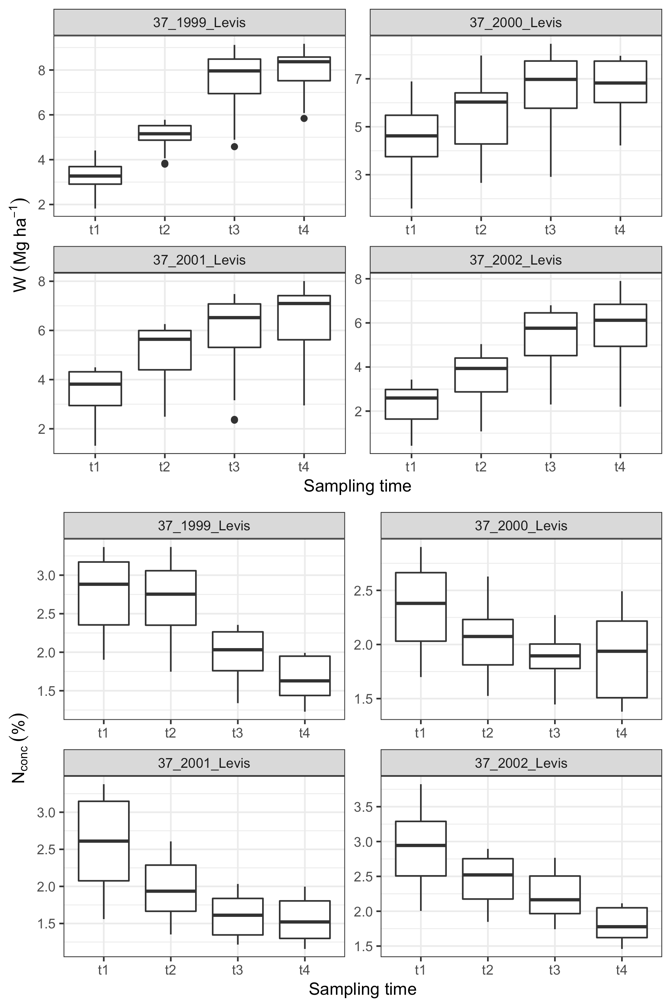


# Wheat


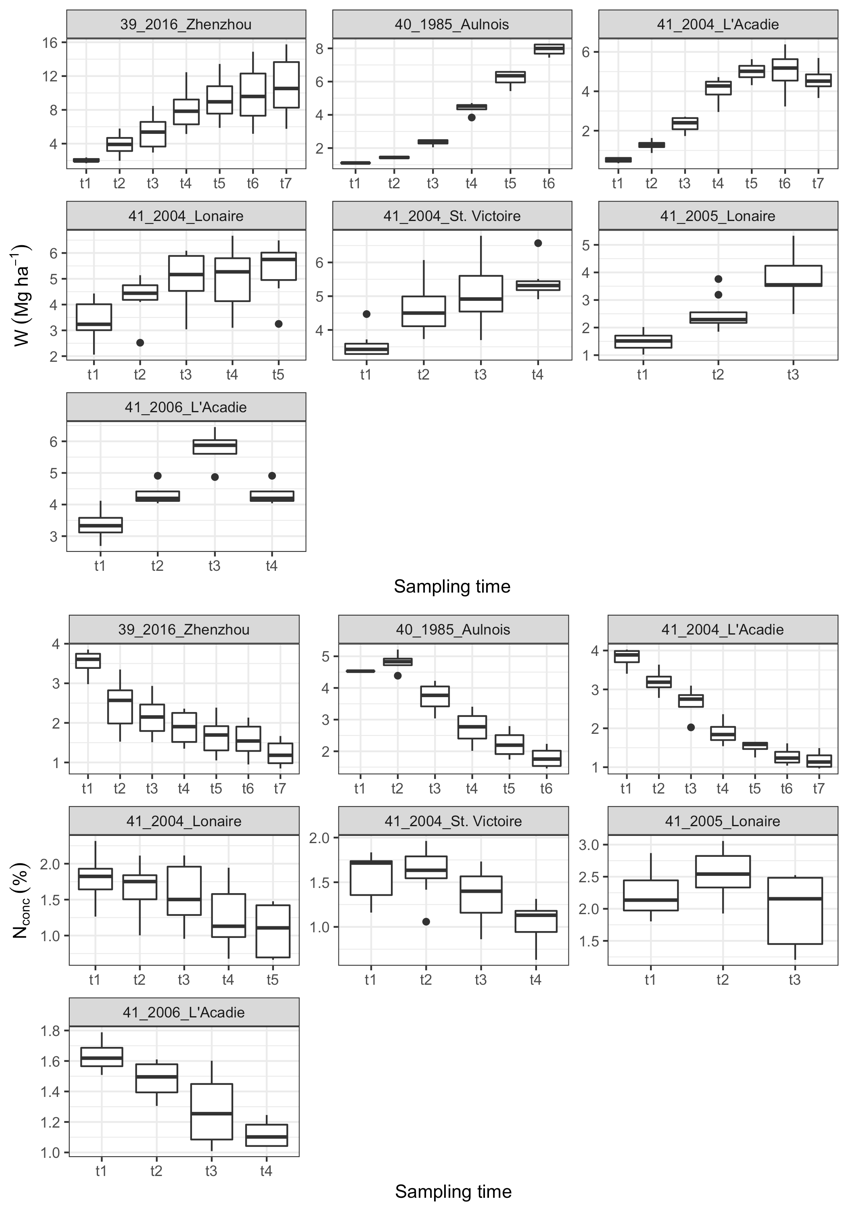


# White cabbage


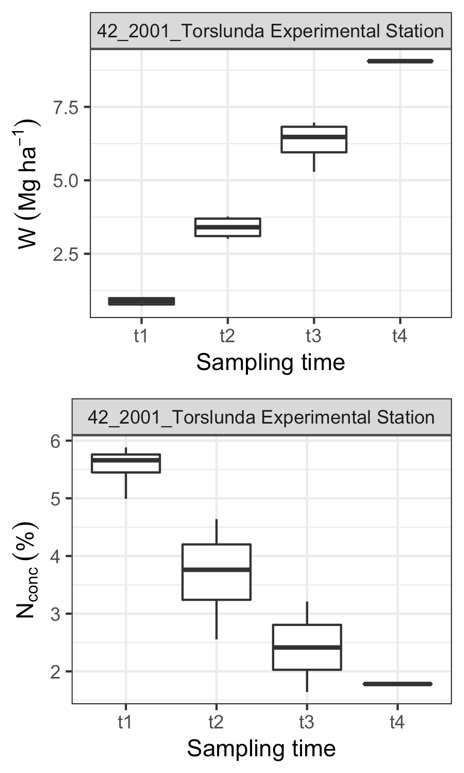

Supplement: Supplementary file 1 — Supplementary Information [file 41597_2022_1395_MOESM1_ESM.docx]
